# Coordinated Expression of Tristetraprolin Post-Transcriptionally Attenuates Mitogenic Induction of the Oncogenic Ser/Thr Kinase Pim-1

**DOI:** 10.1371/journal.pone.0033194

**Published:** 2012-03-08

**Authors:** Dig B. Mahat, Sarah E. Brennan-Laun, Elizabeth J. Fialcowitz-White, Aparna Kishor, Christina R. Ross, Tatyana Pozharskaya, J. David Rawn, Perry J. Blackshear, Bret A. Hassel, Gerald M. Wilson

**Affiliations:** 1 Department of Biochemistry and Molecular Biology, University of Maryland School of Medicine, Baltimore, Maryland, United States of America; 2 Department of Microbiology and Immunology, University of Maryland School of Medicine, Baltimore, Maryland, United States of America; 3 Marlene and Stewart Greenebaum Cancer Center, University of Maryland School of Medicine, Baltimore, Maryland, United States of America; 4 Department of Chemistry, Towson University, Baltimore, Maryland, United States of America; 5 Laboratory of Signal Transduction, NIEHS-NIH, Research Triangle Park, North Carolina, United States of America; Hertie Institute for Clinical Brain Research and German Center for Neurodegenerative Diseases, Germany

## Abstract

The serine/threonine kinase Pim-1 directs selected signaling events that promote cell growth and survival and is overexpressed in diverse human cancers. Pim-1 expression is tightly controlled through multiple mechanisms, including regulation of mRNA turnover. In several cultured cell models, mitogenic stimulation rapidly induced and stabilized *PIM1* mRNA, however, vigorous destabilization 4–6 hours later helped restore basal expression levels. Acceleration of *PIM1* mRNA turnover coincided with accumulation of tristetraprolin (TTP), an mRNA-destabilizing protein that targets transcripts containing AU-rich elements. TTP binds *PIM1* mRNA in cells, and suppresses its expression by accelerating mRNA decay. Reporter mRNA decay assays localized the TTP-regulated mRNA decay element to a discrete AU-rich sequence in the distal 3′-untranslated region that binds TTP. These data suggest that coordinated stimulation of TTP and *PIM1* expression limits the magnitude and duration of *PIM1* mRNA accumulation by accelerating its degradation as TTP protein levels increase. Consistent with this model, *PIM1* and TTP mRNA levels were well correlated across selected human tissue panels, and *PIM1* mRNA was induced to significantly higher levels in mitogen-stimulated fibroblasts from TTP-deficient mice. Together, these data support a model whereby induction of TTP mediates a negative feedback circuit to limit expression of selected mitogen-activated genes.

## Introduction

The *PIM1* gene encodes a serine/threonine kinase that can regulate cell proliferation and survival at multiple levels [1], [2]. For example, Pim-1-mediated phosphorylation of the tyrosine phosphatase Cdc25A increases its activity [3], which includes activation of Cdk2/cyclin E to promote progression from G1 into S phase [4]. In response to genotoxic stress, the cyclin-dependent kinase inhibitor p21^waf/Cip1^ blocks DNA replication by binding to proliferating cell nuclear antigen (PCNA) [5]; however, phosphorylation of p21 by Pim-1 disrupts the p21-PCNA complex, thus stimulating resumption of S phase [6]. Pim-1 activity can also promote progression through the G2/M transition. While phosphorylation of Cdc25C by its associated kinase C-TAK1 blocks the ability of Cdc25C to activate the G2/M switch, phosphorylation of C-TAK1 by Pim-1 abrogates this checkpoint activity [7]. Furthermore, Pim-1 phosphorylation events promote recruitment of nuclear mitotic factors to spindle poles, an essential event in cell division [8]. Beyond enhancing cell proliferation, Pim-1 can also suppress programmed cell death by inactivating the pro-apoptotic proteins Bad [9] and ASK1 [10].

Additional cellular consequences of Pim-1 activity result from its effects on transcriptional control of gene expression. For instance, Pim-1-directed suppression of p27^Kip1^ expression includes inhibition of p27 gene transcription, mediated by phosphorylation and inactivation of the forkhead transcription factors FoxO1a and FoxO3a [11]. Pim-1 also attenuates cytokine-induced transcriptional programs mediated by the JAK-STAT pathways by interacting with the suppressor of cytokine signaling proteins Socs-1 and Socs-3 [12]. Phosphorylation by Pim-1 increases cellular levels of Socs-1 by stabilizing the protein [13], thus enhancing its ability to limit JAK-dependent activation of downstream targets, particularly the transcription factor STAT5 [12]. In a third example, phosphorylation by Pim-1 was shown to activate p100, a transcriptional coactivator that interacts with the transcription factor c-Myb, leading to enhanced transcriptional activation [14]. Finally, Pim-1 can also co-activate MYC-targeted genes, which may involve phosphorylation of proximal histone proteins or even MYC itself [15], [16].

Together, these observations indicate that Pim-1 can profoundly impact cell proliferation and survival, involving direct effects on the cell cycle and apoptotic machinery, as well as indirect effects via re-programming transcriptional regulatory networks. Consistent with this model, overexpressing Pim-1 from an immunoglobulin enhancer induces lymphomas in transgenic mice [17], and elevated Pim-1 levels have been associated with development of hematopoietic cancers as well as aggressive tumors of the stomach and prostate [16], [18]–[21]. Although the consequences of Pim-1 overexpression on cellular growth and survival are severe, cells can normally regulate Pim-1 levels through multiple mechanisms. In hematopoietic cell models, transcription from the *PIM1* gene is dramatically enhanced by a variety of mitogenic stimuli, however, induction is generally transient [22]–[25]. Furthermore, sequences in the 5′-untranslated region (5′UTR) of *PIM1* mRNA can attenuate its translation [26], while turnover of Pim-1 protein is regulated through interactions with heat shock protein 90 and protein phosphatase 2A [27], [28]. An early report characterizing the kinetics of Pim-1 induction indicated that mitogens could also modulate the decay kinetics of *PIM1* mRNA. In primary lymphocytes, treatment with concanavalin A and the phorbol ester 12-*O*-tetradecanoyl-phorbol-13-acetate (TPA) transiently elevated *PIM1* mRNA levels [25]. However, while *PIM1* mRNA was moderately stable when maximally induced, it was destabilized 17 hours following mitogenic stimulation.

Although investigations into the regulation of Pim-1 expression have largely focused on leukocyte models, recent findings that Pim-1 is overexpressed in some non-hematopoietic cancers (above) suggest that mechanisms limiting its induction may be relevant to many different cell types. In this study, we determined that *PIM1* mRNA is rapidly but transiently induced by mitogenic stimulation in cultured human cell models representing three distinct tumorigenic tissues, and in all cases involves rapid but reversible stabilization of *PIM1* mRNA. Destabilization of *PIM1* mRNA several hours after treatment with mitogens was accompanied by dramatically enhanced expression of tristetraprolin (TTP), a tandem CCCH zinc finger protein that targets mRNA substrates for rapid degradation. TTP functions by interacting with several important components of the cytoplasmic mRNA decay machinery, including components of the 5′-decapping complex, 3′-deadenylating complexes, and the 5′→3′ and 3′→5′ exonuclease activities required to degrade the mRNA body [29], [30]. In this work, we also show that TTP binds *PIM1* mRNA in cells and accelerates its decay, and that this post-transcriptional regulatory circuit functions through AU-rich elements (AREs) located near the 3′-end of the transcript. Correlation analyses suggest that expression of TTP and *PIM1* mRNAs are coordinated in diverse cell types. Given recent evidence that a diverse array of mRNAs may associate with and/or be regulated by TTP [31]–[33], we propose that mitogenic induction of TTP serves to attenuate and temporally limit the activation of a subset of mitogen-stimulated genes, including *PIM1*.

## Materials and Methods

### Ethics Statement

All mouse experiments were conducted according to the US Public Health Service policy on the humane care and use of laboratory animals. All animal procedures used in this study were approved by the National Institute of Environmental Health Sciences Institutional Animal Care and Use Committee (protocol number 97-06).

### Cell Culture and Mitogenic Stimulation

MBA-MB-231, HeLa, and HepG2 cells were obtained from the American Type Culture Collection. MDA-MB-231 and HeLa lines were maintained in DMEM+10% fetal bovine serum (FBS) at 37°C and 5% CO_2_ while HepG2 cells were grown in MEM+10% FBS under the same conditions. Primary murine embryonic fibroblasts (MEFs) were isolated from E14.5 embryos of TTP knockout mice (*Zfp*36^−/−^) and wild-type littermates (*Zfp*36^+/+^) as described previously [33] and were maintained in DMEM containing 10% FBS, 100 U/ml penicillin, 100 µg/ml streptomycin, and 2 mM L-glutamine. Experiments employing MEF cultures were performed on cells prior to passage 12. Where indicated, mitogenic stimulation of all cell models was performed by serum starvation in medium containing 0.5% FBS for 16–20 hours, followed by administration of fresh medium containing 10% FBS and 100 nM TPA. HeLa/Tet-Off cell clones stably transfected with expression vectors encoding FLAG-tagged wild type TTP (FLAG-TTPwt) or the TTP C147R mutant (FLAG-C147R) were generated previously [34], and were maintained in DMEM containing 10% FBS, 100 µg/ml G418, 100 µg/ml hygromycin B, and 2 µg/ml doxycycline (Dox). As required, FLAG-TTPwt or FLAG-C147R expression was induced by removal of Dox from growth media for 24 hours.

### Measurements of *PIM1* mRNA Levels and Decay Kinetics

Total RNA was purified from cultured cell lines using TRIzol reagent (Invitrogen) according to the manufacturer's instructions. RNA samples were analyzed for *PIM1* mRNA by qRT-PCR using the iScript One-Step RT-PCR Kit with SYBR Green (Bio-Rad) in parallel reactions programmed with human *PIM1* and GAPDH amplification primers (for MBA-MB-231, HeLa, and HepG2 RNA samples; all qRT-PCR primers are listed in Table S1). Corresponding murine *PIM1* and GAPDH PCR primers were used for RNA samples from MEF cultures. Relative levels of *PIM1* mRNA were calculated from threshold cycle numbers (*Ct*) after normalization to endogenous GAPDH mRNA abundance using the 2^ΔΔ*Ct*^ method. Each data point was taken as the mean ± standard deviation from quadruplicate qRT-PCR reactions for each RNA sample. The decay kinetics of *PIM1* mRNA was measured by actinomycin D (actD) time course assay. Briefly, total RNA samples were purified from cultured cells at various times following treatment with actD (5 µg/ml), which inhibits global transcription. Time courses were limited to 4 h to avoid complicating cellular mRNA decay pathways by actD-enhanced apoptosis [35]. Relative *PIM1* mRNA levels remaining at each time point were quantified by qRT-PCR (described above), normalized to GAPDH mRNA, and plotted as a function of time following actD treatment. From these plots, first-order mRNA decay constants (*k*) were resolved by nonlinear regression (PRISM v3.03, GraphPad), from which *PIM1* mRNA half-lives were calculated using *t*_1/2_ = ln2/*k*. Tabulated *PIM1* mRNA half-life values are based on the mean ± standard deviation of *n* independent time-course experiments to permit pair-wise statistical comparisons (described below).

### β-globin Reporter mRNA Decay Assays

The effects of *PIM1* mRNA 3′UTR sequences on TTP-directed mRNA decay were analyzed using β-globin (βG)-chimeric transcripts essentially as described [36]. Briefly, selected sequences were amplified by PCR from a *PIM1* cDNA clone (GenBank accession NM_002648; GeneCopoeia) using *Pfu* DNA polymerase. A *PIM1* cDNA fragment encoding a mutated ARE domain was synthesized by GenScript. These fragments were subcloned downstream of the βG translational termination codon in vector pTRERβ, which expresses the rabbit βG gene under the control of a tetracycline-responsive promoter [37]. The fidelity of all recombinant plasmids was verified by restriction mapping and automated DNA sequencing. Reporter plasmids (50 ng) were transfected into HeLa/Tet-Off cells (Clontech) in 6-well plates along with the control plasmid pEGFP-C1 (200 ng; Clontech), encoding the enhanced green fluorescent protein (EGFP), using Superfect reagent (Qiagen). Where indicated, cells were cotransfected with vectors (100 ng) expressing FLAG-TTPwt or FLAG-TTP C147R from constitutive promoters, or with an empty vector (pcDNA) as a negative control. After 24 h, transcription from the βG reporter plasmids was arrested by adding doxycycline (Dox; 2 µg/ml). At selected time points thereafter, DNA-free RNA was harvested using the SV RNA Purification Kit (Promega) and analyzed for βG-reporter and EGFP mRNA levels by multiplex qRT-PCR using the qScript One-Step qRT-PCR Kit (Quanta Biosciences) with βG and EGFP Taqman primer/probe sets (Table S1) as described previously [36], with each data point taken as the mean ± standard deviation of five qRT-PCR reactions. After normalization to EGFP mRNA concentrations, the levels of individual βG-reporter mRNAs were plotted as a function of time following administration of Dox to resolve mRNA decay constants as described above.

### Western Blots

Rabbit anti-TTP was from Abcam. Rabbit anti-Pim-1, mouse anti-FLAG M2 monoclonal, horseradish peroxidase-conjugated anti-GAPDH, and all secondary antibodies were from Sigma. Whole cell lysates were collected by washing cell monolayers with phosphate-buffered saline and then scraping in 2× SDS-PAGE buffer (250 mM Tris [pH 6.8] containing 2% SDS, 10 mM DTT, 10% glycerol, and 0.05% bromophenol blue). Cell lysates were heated to 100°C for 5 min, then clarified by centrifugation at 16,000× *g* for 10 min to precipitate cell debris. Clarified lysates were fractionated through 10% SDS polyacrylamide gels and transferred to a nitrocellulose membrane which was then blocked with 10% nonfat milk and incubated overnight with primary antibody at 4°C. After washing, blots were then incubated with peroxidase-conjugated secondary antibodies for an hour and developed using the Western Lightning Plus-ECL kit (PerkinElmer).

### Immunoprecipitation and RT-PCR of Ribonucleoprotein Complexes

HeLa cells were lysed in PLB buffer (10 mM HEPES [pH 7.5] containing 100 mM KCl, 5 mM MgCl_2_, 0.5% IGEPAL CA630, and 1 mM dithiolthreitol) containing 250 U/ml RNaseOUT (Invitrogen) and 1× complete protease inhibitor cocktail (Roche) on ice for 10 minutes. Ribonucleoprotein (RNP) complexes containing FLAG-TTPwt or FLAG-TTP C147R were fractionated from these lysates by incubation with 100 µl of a 50% (v/v) suspension of Protein-A Sepharose beads (Sigma) pre-coated with 30 µg M2 anti-Flag monoclonal antibody (Sigma) for 2 h at 4°C with mixing. Parallel fractionations programmed with mouse IgG1- (BD Pharmingen) loaded beads served as negative controls. After incubation beads were washed 5 times with NT2 buffer (50 mM Tris [pH 7.4] containing 150 mM NaCl, 1 mM MgCl_2_, 0.05% Triton X-100), and then incubated with 100 µl NT2 buffer containing RNase-free DNase I (20 U) for 15 min at 30°C to eliminate DNA from samples. Subsequently, beads were washed twice with 1 ml NT2 buffer, and then incubated in 100 µl NT2 buffer containing 0.1% SDS and 0.5 mg/ml proteinase K for 15 min at 55°C to digest proteins bound to the beads. After extraction with phenol∶chloroform (1∶1), the RNA from each ribonucleoprotein immunoprecipitation (RNP-IP) was then reverse-transcribed and specific transcripts quantified using the iScript One-step RT-PCR SYBR Green kit (Bio-Rad) with primer sets listed in Table S1.

### Biotin-RNA Pull-down Assay

Interactions between FLAG-TTP proteins expressed in HeLa cells and RNA substrates were evaluated *in vitro* using a modification of the biotin-RNA pull-down assay described by Wang *et al.* [38]. Briefly, *in vitro* transcription templates encoding the *PIM1* ARE, a mutated ARE fragment, or a coding region sequence from *PIM1* mRNA downstream of the T7 promoter were generated by PCR using *Pfu* DNA polymerase (Stratagene) from appropriate primers. Biotin-labeled riboprobes were then generated using the MegaShortScript T7 *in vitro* transcription kit (Ambion) incorporating UTP and biotin-16-UTP (Roche) at a 9∶1 ratio. Crude cytoplasmic extracts were prepared from HeLa/Tet-Off cells or clonal lines expressing FLAG-TTPwt or FLAG-TTP C147R by scraping into lysis/wash buffer (10 mM TrisHCl [pH 7.5] containing 100 mM KCl, 2.5 mM MgCl_2_, 2 mM dithiolthreitol, and 1% IGEPAL-CA630) supplemented with a protease inhibitor cocktail (1 µg/ml leupeptin, 1 µg/ml pepstatin A, and 0.1 mM phenylmethylsulfonyl fluoride). Cells were broken using a Dounce homogenizer and nuclei pelleted by centrifugation at 1000× *g* for 10 minutes. Protein concentrations were measured using the Bio-Rad Protein Assay reagent. Biotin-RNA pull-down reactions were assembled with 50 µg protein extract and 20 pmol biotin-RNA. After incubation for 30 minutes at room temperature, biotin-RNA:protein complexes were isolated using streptavidin-agarose beads (Fluka), washed twice in lysis/wash buffer, then dissociated by re-suspension in 2× SDS-PAGE buffer at 100°C for 5 minutes. Co-purification of FLAG-tagged TTP proteins was determined using Western blots.

### Statistics

Comparisons of mRNA levels and decay kinetics were done using the unpaired *t* test, while correlation analyses used the Spearman nonparametric test. In all cases, differences yielding *p*<0.05 were considered significant.

## Results

### Transient Mitogenic Stimulation of *PIM1* Expression Includes Reversible mRNA Stabilization in Diverse Human Cultured Cell Models

**Figure 1:**
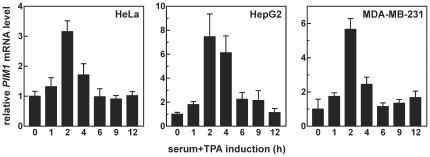
Transient induction of endogenous *PIM1* mRNA by mitogenic stimuli in cancer cell lines. Total RNA was isolated from HeLa, HepG2, and MDA-MB-231 cells at selected times following stimulation with serum+TPA as described in “Materials and Methods”. Bars represent the relative levels of *PIM1* mRNA determined by qRT-PCR and normalized to GAPDH mRNA (mean ± SD of quadruplicate qRT-PCR reactions). Independent replicate experiments yielded similar results.

Previous studies showed that mitogens can transiently induce *PIM1* gene transcription in a variety of hematopoietic cell models (described under Introduction), however, few details are available regarding the regulatory mechanisms responsible for temporal control of *PIM1* expression. Furthermore, little is known about the regulation of *PIM1* expression in non-hematopoietic cells, even though it is overexpressed in some solid tumors. The report by Wingett *et al.* [25] raised the interesting possibility that the diminution of *PIM1* mRNA that followed its induction by mitogens in primary lymphocytes was accompanied by destabilization of the transcript. In order to characterize molecular events contributing to transient accumulation of *PIM1* mRNA, and to ascertain whether these mechanisms also applied to non-hematopoietic cell types, it was first necessary to determine whether *PIM1* mRNA was regulated by mitogenic stimulation in tractable cultured cell systems. To this end, we monitored *PIM1* mRNA levels in serum-starved HeLa (human cervical adenocarcinoma), HepG2 (human hepatoblastoma), and MDA-MB-231 (human breast adenocarcinoma) cells, then measured changes in *PIM1* mRNA expression as a function of time following mitogenic stimulation using serum+TPA. In all three cell models, *PIM1* mRNA was significantly induced 2 hours following stimulation, but returned to near basal levels shortly thereafter (Figure 1).

**Figure 2:**
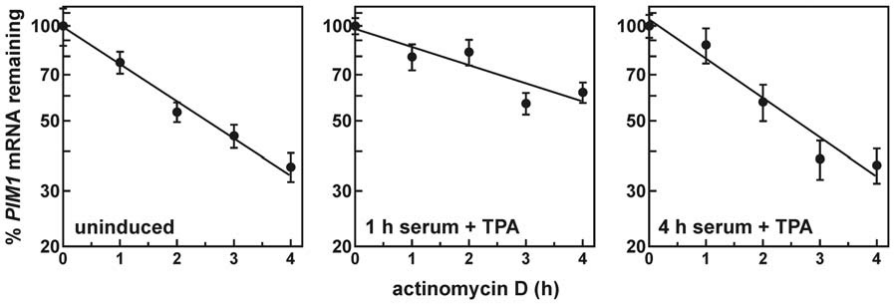
Control of *PIM1* mRNA turnover in mitogen-stimulated HeLa cells. The decay kinetics of *PIM1* mRNA were measured in serum-starved HeLa cells (uninduced) or at selected times after stimulation with serum+TPA using actD time course assays. For each experiment, the fraction of *PIM1* mRNA remaining was plotted as a function of time following inhibition of transcription by actD, and *PIM1* mRNA decay constants resolved by nonlinear regression to a first-order decay model (*lines*). Average decay constants measured across replicate independent experiments are listed in Table 1.

To determine whether mitogen-induced changes in *PIM1* mRNA levels included modulation of mRNA turnover kinetics, actD time course assays were used to measure *PIM1* mRNA decay rates in cells prior to or at selected times following mitogenic stimulation. In HeLa cells, *PIM1* mRNA decay was well described by a first-order kinetic model, which in uninduced cells yielded an mRNA half-life of approximately 2.4 hours (Figure 2 and Table 1). One hour following application of serum+TPA, *PIM1* mRNA was stabilized greater than 2-fold. However, this mitogen-induced inhibition of *PIM1* mRNA decay was reversed 4 hours following stimulation of HeLa cells (Table 1), concomitant with decreasing levels of the *PIM1* transcript (Figure 1). In HepG2 and MDA-MB-231 cells, similar trends in *PIM1* mRNA decay kinetics were observed, although the stabilization phase was even more pronounced, with *PIM1* mRNA exhibiting a half-life of >10 hours following 1 hour serum+TPA treatment (Table 1). These data indicate that mitogenic stimulation quickly stabilizes *PIM1* mRNA in concert with the previously described activation of *PIM1* gene transcription [22]–[24], [39]. However, following this transient accumulation phase *PIM1* mRNA is destabilized, which likely accelerates the rate at which *PIM1* mRNA returns to basal levels in the cell. Finally, these data show that this reversible mRNA stabilization event occurs in a wide range of cell types.

**Table 1:** *PIM1* mRNA decay kinetics during mitogenic stimulation of cancer cell lines.

cell line	serum+TPA^a^	*t*_1/2_ (h)^b^	*n*
**HeLa**	unstimulated	2.38±0.16	3
	1 h	5.27±0.15	3
	4 h	2.33±0.09	3
**HepG2**	unstimulated	1.63±0.12	3
	1 h	>10	3
	4 h	2.68±0.21	3
**MDA-MB-231**	unstimulated	3.05±0.20	3
	1 h	>10	3
	4 h	3.36±0.58	3

a Cultures were incubated for 16–20 h in medium containing 0.5% serum prior to each experiment. Where indicated, cells were stimulated by adding medium containing serum (10%) and TPA (100 nM) for indicated periods prior to inhibition of transcription with actD.

b First-order mRNA decay constants (*k*) were resolved for each cell population by actD time course assay as described under “Materials and Methods”. mRNA half-lives were then calculated using *t*_1/2_ = ln2/*k*. Quoted values represent the mean ± SD across *n* independent time course experiments.

### Post-mitogen Suppression of *PIM1* mRNA Coincides with Induction of TTP, which Binds and Destabilizes the *PIM1* Transcript

**Figure 3:**
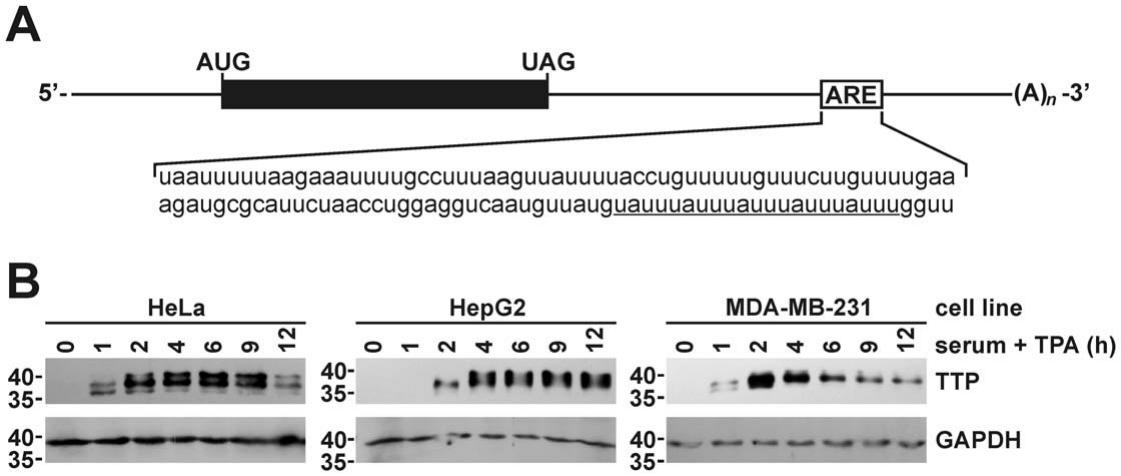
Induction of the ARE-binding protein TTP in mitogen-stimulated cancer cell lines. (A) Schematic of *PIM1* mRNA showing a putative ARE sequence within the distal 3′UTR. Sequences corresponding to consensus TTP-binding sites are underlined. (B) Western blots showing the induction of TTP protein at selected times following mitogenic stimulation of cancer cell lines. Whole cell lysates were prepared at indicated time points following addition of serum+TPA to serum-starved cultures. GAPDH levels were used to normalize protein loading. The positions of molecular weight markers (in kDa) are indicated left of each blot.

Regulated mRNA decay is generally directed by discrete *cis*-acting sequences within affected transcripts. The best characterized sequence determinants of mRNA stability are AREs, which are located within the 3′UTRs of many mRNAs that encode oncoproteins and inflammatory mediators [40]. AREs function by associating with cellular ARE-binding proteins, which may positively or negatively influence mRNA decay rates or translational efficiency [41], [42]. Towards the 3′-end of the *PIM1* mRNA 3′UTR is a U-rich domain containing several overlapping copies of the AUUUA motif common among ARE sequences (Figure 3A). A further indication that this domain might contribute to the regulated decay of *PIM1* mRNA was previously reported, as a germ-specific *PIM1* transcript found in rat testes which lacks the distal 3′UTR is significantly more stable than the somatic *PIM1* mRNA [25]. Although many different factors can influence mRNA decay kinetics through AREs, two observations suggested that the ARE-binding, mRNA-destabilizing factor TTP might contribute to the regulated decay of *PIM1* mRNA following mitogenic stimulation. First, *PIM1* mRNA levels were suppressed 2–4 hours following stimulation with serum+TPA in several cultured cell models (Figure 1) involving destabilization of *PIM1* mRNA (Figure 2), while TTP expression is induced by mitogenic stimuli in some cell types [43], [44]. Second, the ARE-like domain within the *PIM1* mRNA 3′UTR contains several sequences of the type UUAUUUAUU (Figure 3A), which were previously identified as high affinity TTP binding sites [45]. Together, these observations raise the possibility that mitogen-stimulated production of TTP might be responsible for limiting expression of *PIM1* mRNA once TTP protein has accumulated in the cell.

**Figure 4:**
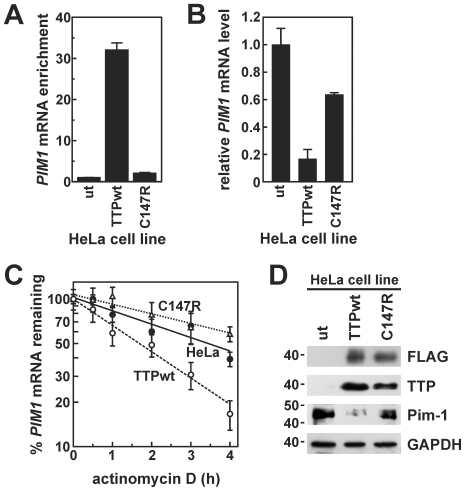
Functional association of TTP with *PIM1* mRNA in HeLa cells. (A) RNP-IP experiments were performed using control IgG or anti-FLAG antibodies and lysates from untransfected HeLa/Tet-Off cells (ut) or stable clonal lines expressing FLAG-TTPwt or FLAG-TTP C147R as described in “Materials and Methods”. Immunoprecipitated material was then screened for *PIM1* mRNA by quantitative real-time RT-PCR and normalized to GAPDH mRNA (mean ± SD of three reactions). Independent replicate experiments yielded similar results. (B) Relative levels of *PIM1* mRNA were measured in untransfected *versus* FLAG-TTPwt- or FLAG-TTP C147R-expressing HeLa cells. Bars represent the mean ± SD of quadruplicate qRT-PCR reactions normalized to GAPDH mRNA. (C) The decay kinetics of *PIM1* mRNA was measured in HeLa cell models using actD time course assays as described in Figure 2. mRNA half-lives calculated from independent replicate experiments are provided in the text. (D) Western blot analyses using antibodies targeting specified proteins in HeLa/Tet-Off cell models, with positions of molecular weight markers (in kDa) shown at left.

To test this model, we first used Western blots to assess TTP protein levels in each cell model as a function of time following mitogenic stimulation. Previously, we and others have shown that TTP is very weakly expressed in a variety of exponentially growing cultured cancer cell lines including HeLa and MDA-MB-231 [34], [46]. Similarly, we observed that TTP protein was barely detectable in serum-starved HeLa, HepG2, or MDA-MB-231 cells (Figure 3B). However, TTP expression was dramatically enhanced in each of these cell models following addition of serum+TPA. TTP protein reached peak levels within 2–4 hours following mitogenic stimulation depending on cell type. In HeLa and MDA-MB-231 cells, TTP protein levels then decreased as a function of time, while in HepG2 cells high TTP expression was maintained for at least 12 hours. At later time points slower mobility bands appeared on TTP immunoblots consistent with post-translationally modified proteins. These modifications are likely phosphorylation events; TTP phosphorylation by the p38^MAPK^-activated kinase MK2 has been shown to regulate both the stability and subcellular distribution of the protein [29]. However, since post-mitogen destabilization of *PIM1* mRNA (4 h post-induction; Table 1) was observed concomitant with dramatically elevated TTP expression, we next tested whether TTP could interact with endogenous *PIM1* transcripts. For these experiments, we utilized previously described HeLa/Tet-Off cell models that express FLAG-tagged versions of wild type TTP (FLAG-TTPwt) or the TTP C147R mutant protein under the control of a tetracycline-regulated promoter [34]. The C147R mutant protein serves as a negative control, since disruption of this Zn^2+^-coordinating residue within the C-terminal zinc finger domain abrogates RNA-binding activity [47]. In RNP-IP assays programmed with anti-FLAG antibodies, *PIM1* mRNA was readily detected in immunoprecipitates from cells expressing wild type TTP but not from untransfected cells or those expressing the C147R mutant (Figure 4A), indicating that *PIM1* mRNA selectively associates with the wild type FLAG-TTP protein.

Given that TTP can interact with *PIM1* mRNA, the next objective was to determine whether TTP influences the expression of this transcript in cells. Real-time qRT-PCR assays showed that *PIM1* mRNA levels were suppressed by over 80% in HeLa/Tet-Off cells expressing FLAG-TTPwt relative to untransfected cells and 70% relative to C147R-expressing cells (Figure 4B), indicating that maximal suppression of *PIM1* mRNA occurs only in the presence of functional TTP. Since TTP normally enhances degradation of substrate mRNAs [48], we then used actD time course assays to determine whether FLAG-TTPwt suppresses *PIM1* mRNA levels by accelerating its decay kinetics (Figure 4C). In untransfected HeLa/Tet-Off cells, *PIM1* mRNA decayed with a half-life of 3.04±0.36 h (*n* = 3). In cells expressing FLAG-TTP C147R, *PIM1* mRNA was slightly more stable (*t*_1/2_ = 4.59±0.85 h; *n* = 4), a small but statistically significant (*p* = 0.033) effect that may reflect a dominant negative activity by the C147R protein on cellular mRNA decay kinetics. Other RNA binding-defective TTP mutants are known to behave similarly [32], [47], possibly as a result of sequestering ancillary mRNA-degrading activities that bind flanking TTP protein domains [49], [50]. Curiously, *PIM1* mRNA levels were modestly decreased in C147R-expressing relative to untransfected cells (Figure 4B), despite being slightly more stable in the C147R line. One possibility is that C147R-induced perturbations in the cellular mRNA decay machinery indirectly contribute to a slight decrease in the transcription of *PIM1* (and likely many other) genes, although through an unknown mechanism. However, in cells expressing FLAG-TTPwt, *PIM1* mRNA decayed with a half-life of 1.73±0.18 h (*n* = 4), which was significantly faster than the turnover rate of this transcript in either untransfected (*p* = 0.0013) or C147R-expressing cells (*p* = 0.0006). Together, these data show that wild type TTP can associate with the *PIM1* transcript in cells, and that this interaction decreases *PIM1* mRNA levels by accelerating its decay. Finally, accelerated decay of *PIM1* mRNA by TTP also impacts levels of the encoded protein, since Western blots show a dramatic decrease in Pim-1 protein in HeLa/Tet-Off cells expressing FLAG-TTPwt relative to untransfected cells (Figure 4D). Consistent with comparisons of *PIM1* mRNA (Figure 4B), expression of the TTP C147R mutant also decreased Pim-1 protein levels modestly, however, they remained substantially higher than in cells expressing comparable amounts of wild type TTP.

### TTP Binds and Destabilizes *PIM1* mRNA via AU-rich Sequences in its Distal 3′UTR

**Figure 5:**
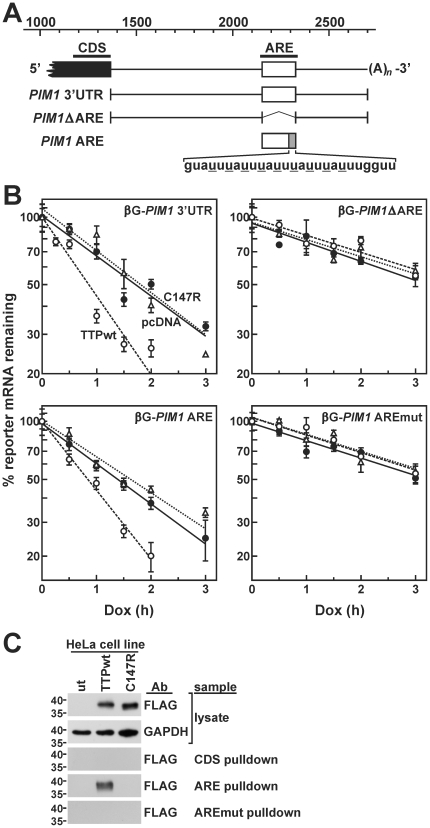
Localization of TTP-responsive elements to an ARE-like sequence in the *PIM1* mRNA 3′UTR. (A) Schematic of the 3′-end of the *PIM1* mRNA coding sequence (*black box*) and complete 3′UTR, including the ARE domain (*white box*). The scale bar (top) is relative to the translational initiation codon. The positions of biotin-labeled riboprobes corresponding to the *PIM1* ARE and coding sequence fragment (CDS) are shown as black bars above the mRNA schematic. Bars below delineate *PIM1* 3′UTR domains that were subcloned downstream of the translational termination codon of the βG gene for reporter mRNA decay assays. At the bottom is the sequence at the extreme 3′-end of the ARE domain that contains known high affinity TTP-binding motifs. In the βG-*PIM1* AREmut reporter mRNA and biotin-labeled AREmut RNA probe, these motifs were disrupted by mutating underlined uridylate residues to cytidines. (B) Decay rates of βG-*PIM1* chimeric reporter mRNAs were resolved by Dox time course assays in HeLa/Tet-Off cells co-transfected with an empty vector (pcDNA; *solid circles*, *solid lines*) or vectors expressing FLAG-TTPwt (*open circles*, *dashed lines*) or FLAG-TTP C147R (*triangles*, *dotted lines*) as described under “Materials and Methods”. mRNA half-lives resolved from multiple independent experiments are summarized in Table 2. (C) Western blots probed with indicated antibodies (Ab) show levels of FLAG-TTP wt and C147R mutant proteins (top panel) and GAPDH (second panel) in crude cytoplasmic extracts prepared from untransfected HeLa/Tet-Off cells (ut) or stable clonal lines expressing each FLAG-TTP variant. Samples of each lysate were fractionated using biotin-RNA pull-down assays programmed with riboprobes encoding a *PIM1* coding sequence fragment (CDS), the *PIM1* ARE or the ARE mutant containing the U→C substitutions specified above (AREmut). FLAG-TTP proteins co-purifying with each riboprobe were detected by Western blot (bottom panels). The positions of molecular weight markers (in kDa) are shown to the left of each Western blot panel.

TTP is known to target a variety of ARE-containing mRNAs, particularly those that encode cytokines and lymphokines [31]. Furthermore, *in vitro* binding studies identified UUAUUUAUU as a high affinity TTP-binding motif [45], several copies of which are localized to the distal 3′UTR of *PIM1* mRNA (Figure 3A). To determine whether this ARE-like domain within the *PIM1* 3′UTR was involved in TTP-directed control of mRNA decay, a series of *PIM1* 3′UTR-derived fragments were inserted into the 3′UTR of a β-globin (βG) reporter gene downstream of a Tet-responsive promoter (Figure 5A). These vectors were co-transfected along with plasmids expressing wild type or C147R mutant forms of FLAG-TTP into HeLa/Tet-Off cells, permitting measurement of reporter mRNA decay rates using Dox time course assays. A βG reporter mRNA containing the entire *PIM1* 3′UTR decayed with a half-life of approximately 1.6 hours in HeLa/Tet-Off cells when co-transfected with an empty control vector (Figure 5B). In cells expressing wild type FLAG-TTP, this reporter transcript decayed with a half-life of 56 minutes, significantly faster than in cells co-transfected with either control (*p* = 0.0006) or C147R-expressing (*p* = 0.0003) plasmids (Table 2). By contrast, a reporter mRNA lacking the *PIM1* ARE (ΔARE) exhibited similar decay kinetics in the presence or absence of functional TTP, indicating that the ARE domain is required for TTP-directed control of mRNA turnover. This was further supported by decay of a βG reporter mRNA containing the *PIM1* ARE alone, which was significantly destabilized in cells expressing wild type TTP relative to cells co-transfected with the C147R mutant (*p* = 0.0087) or empty vector control (*p* = 0.0094). Finally, we tested whether the UUAUUUAUU sequences located at the 3′-end of the ARE domain specifically contributed to TTP-dependent mRNA destabilization by measuring the decay kinetics of a modified βG-*PIM1* ARE reporter transcript (βG-*PIM1* AREmut) containing a series of U→C substitutions within these motifs (Figure 5A). Similar to the *PIM1* ΔARE reporter, turnover of the βG-*PIM1* AREmut mRNA was completely unresponsive to TTP expression (Table 2), indicating that the UUAUUUAUU motifs located within the distal portion of the ARE domain are essential for targeted mRNA decay through TTP.

**Table 2:** Decay kinetics of βG-*PIM1* chimeric mRNAs in transfected HeLa cells.

mRNA	Co-transfection	*t*_1/2_ (h)^a^	*n*
**βG-** ***PIM1*** ** 3′UTR**	pcDNA	1.64±0.09	3
	TTPwt	0.93±0.09	3
	TTP C147R	1.66±0.11	4
**βG-** ***PIM1*** ** ΔARE**	pcDNA	3.88±0.71	3
	TTPwt	3.67±0.22	3
	TTP C147R	4.52±0.49	4
**βG-** ***PIM1*** ** ARE**	pcDNA	1.42±0.07	3
	TTPwt	0.89±0.18	3
	TTP C147R	1.45±0.09	3
**βG-** ***PIM1*** ** AREmut**	pcDNA	2.58±0.56	4
	TTPwt	2.96±0.54	4
	TTP C147R	2.93±0.17	3

a First-order constants (*k*) describing the decay kinetics of indicated βG-chimeric mRNAs were measured in HeLa/Tet-Off cells co-transfected with indicated expression plasmids by Dox time course assays as described under “Materials and Methods” and in Figure 5. mRNA half-lives were calculated using *t*_1/2_ = ln2/*k*. Quoted values represent the mean ± SD for *n* independent experiments.

To determine whether TTP could physically interact with the ARE from *PIM1* mRNA, biotin-labeled riboprobes were synthesized that encoded a 171-nucleotide region spanning the *PIM1* ARE or a comparably sized fragment from the 3′-end of the *PIM1* coding sequence (Figure 5A). When incubated with crude cytoplasmic extracts from untransfected HeLa/Tet-Off cells or cultures expressing FLAG-TTPwt or FLAG-TTP C147R, the wild type protein co-purified with the biotin-labeled ARE fragment over a streptavidin resin, while the mutant protein did not (Figure 5C). Neither FLAG-TTP protein co-purified with the *PIM1* mRNA coding sequence fragment. Similarly, neither protein was recovered in complexes with the biotin-labeled *PIM1* AREmut probe. Together, these data demonstrate that the UUAUUUAUU-enriched sequences at the 3′-end of the ARE-like domain within the *PIM1* 3′UTR bind TTP, and are required for acceleration of mRNA decay in the presence of this factor.

### Expression of *PIM1* and TTP mRNAs are Coordinately Regulated in Various Tissues

**Figure 6:**
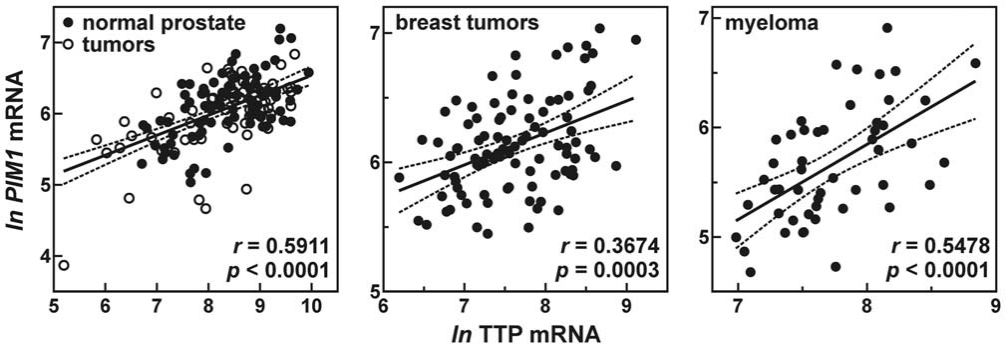
Coordinated expression of *PIM1* and TTP mRNAs in selected human tissues. Levels of *PIM1* and TTP mRNAs were extracted from gene array datasets derived from a panel of human non-transformed (normal) and tumorous prostate tissues (Gene Expression Omnibus accession no. GSE6919), breast tumors (Gene Expression Omnibus accession no. GSE2294), and CD138+ cells purified from bone marrow of multiple myeloma (MM) patients (Gene Expression Omnibus accession no. GSE2912). Correlation between levels of *PIM1* and TTP mRNAs was analyzed using the Spearman nonparametric test. Correlation coefficients (*r*) and associated *p* values are listed in each panel. The dotted lines indicate the 95% confidence intervals of each regression solution.

Our working model is that mitogenic stimulation concomitantly induces expression of both *PIM1* (Figure 1) and TTP (Figure 3B), and that the resulting enhancement of TTP protein serves to limit the amplitude and duration of *PIM1* mRNA accumulation by targeting this transcript for degradation. While this relationship was consistent among the cultured cell models surveyed in this work, we next tested whether *PIM1* and TTP expression might be coordinately regulated *in vivo* by comparing *PIM1* and TTP mRNA levels among gene array datasets derived from cohorts of human tissues (Figure 6). The datasets tested represented: (i) a collection of 171 prostate samples, which included normal and transformed tissues [51], (ii) 94 breast tumors [52], and (iii) CD138+ cells purified from the bone marrow of 50 multiple myeloma (MM) patients [53]. In all cases, statistically significant positive correlations were observed between *PIM1* and TTP mRNA levels. If both *PIM1* and TTP were constitutively expressed, one would expect a negative correlation between these mRNAs, since the steady-state level of *PIM1* mRNA would be suppressed by TTP-directed destabilization. However, since both are inducible genes, these data are most consistent with a model whereby *PIM1* and TTP expression are concomitantly induced by common stimuli, and that this relationship is conserved across diverse tissue types.

### Coordinated Induction of *PIM1* and TTP Limits the Magnitude and Duration of *PIM1* mRNA Accumulation Following Mitogenic Stimulation

**Figure 7:**
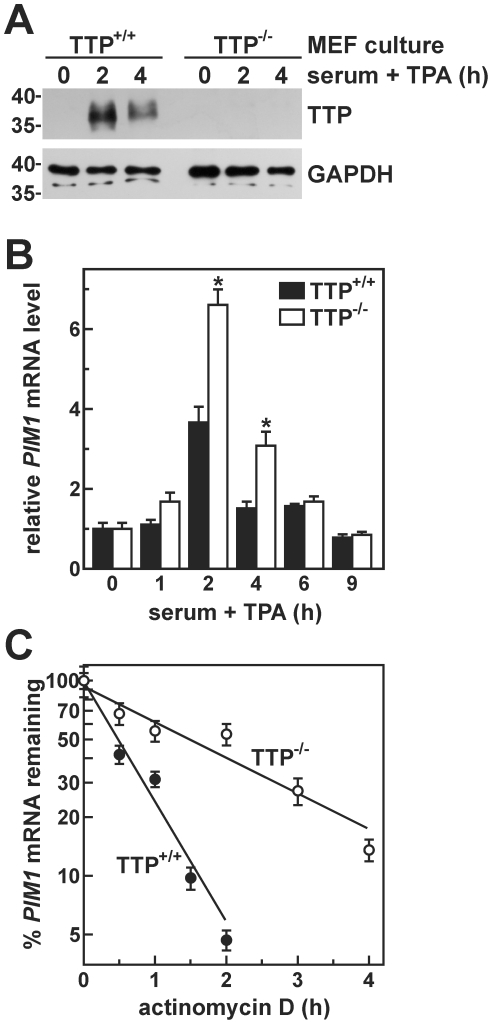
Regulation of *PIM1* mRNA induction by TTP following mitogenic stimulation in MEF models. (A) Whole cell lysates were prepared from MEFs derived from TTP knockout mice (TTP^−/−^) and wild-type littermates (TTP^+/+^) following serum-starvation and stimulation with serum+TPA as described in “Materials and Methods”. Expression of TTP and GAPDH were assessed at selected time points by Western blot, with the positions of molecular weight markers (in kDa) shown at left. (B) Total RNA was isolated from MEF cultures stimulated as described in (A). Bars show the relative level of *PIM1* mRNA in TTP^+/+^ (*solid bars*) and TTP^−/−^ (*open bars*) MEFs at indicated times following mitogenic stimulation as determined by qRT-PCR and normalized to GAPDH mRNA (mean ± SD of quadruplicate qRT-PCR reactions, *p<0.01 *versus* TTP^+/+^). Independent replicate experiments yielded similar results. (C) ActD was added to MEF cultures 2 hours after stimulation with serum+TPA. *PIM1* mRNA decay rates were then measured as described in Figure 2, and yielded half-lives of 0.42±0.11 h (*n* = 4) for TTP^+/+^ cells *versus* 1.56±0.12 h (*n* = 3) for TTP^−/−^ (*p*<0.0001 *versus* TTP^+/+^).

Finally, to test whether mitogenic induction of TTP is required to attenuate *PIM1* expression in mitogen-stimulated cells, *PIM1* mRNA levels were compared in MEF cultures derived from TTP knockout mice (TTP^−/−^) *versus* wild type littermates (TTP^+/+^) as a function of time following treatment with serum+TPA. Similar to the cultured human cell lines (Figure 3B), mitogenic stimulation rapidly and potently increased TTP protein levels in TTP^+/+^ MEFs (Figure 7A), while no TTP protein was detected in the TTP^−/−^ line. In TTP^+/+^ cells, *PIM1* mRNA was rapidly but transiently induced following addition of serum+TPA, increasing approximately 3.5-fold within 2 hours (Figure 7B). By contrast, mitogenic stimulation of TTP^−/−^ MEFs increased *PIM1* mRNA to levels 1.8-fold higher than those observed in the corresponding TTP^+/+^ line. Furthermore, enhanced *PIM1* levels were observed for a longer period following stimulation of TTP^−/−^ *versus* TTP^+/+^ MEFs. Four hours post-stimulation, *PIM1* mRNA was still elevated 3-fold above uninduced levels in TTP^−/−^ cells, while in cells expressing TTP, *PIM1* mRNA had returned to near basal levels at this point. Finally, ActD time course assays performed after 2 h induction showed that PIM1 mRNA decayed over 3.5-fold faster in TTP^+/+^ *versus* TTP^−/−^ MEFs (Figure 7C). Together, these data show that concomitant induction of TTP limits the accumulation of *PIM1* mRNA following mitogenic stimulation by accelerating decay of this transcript.

## Discussion

Mitogens rapidly induce expression from the *PIM1* gene in many different cell backgrounds (Figure 1) [23]–[25]. The resulting enrichment of Pim-1 protein levels activates several nuclear and cytoplasmic signaling programs that promote cell proliferation and suppress apoptosis (described under “Introduction”). However, prolonged or constitutive elevation of Pim-1 levels can contribute to hyperproliferative or neoplastic syndromes [16], [17], [20], [21], indicating that it is essential to restrict *PIM1* expression. In this study, we show that induction of *PIM1* mRNA following mitogenic stimulation with serum+TPA is temporally limited in several cell models (Figure 1), and that rapid restoration to basal expression levels involves acceleration of mRNA decay in each case (Table 1). This post-induction enhancement of *PIM1* mRNA turnover coincides with accumulation of the ARE-binding protein TTP (Figure 3), which binds and destabilizes *PIM1* mRNA (Figure 4) *via* a series of UUAUUUAUU motifs located within an ARE-like domain in the *PIM1* 3′UTR (Figure 5). Finally, we provide evidence that expression of TTP and *PIM1* are correlated in many human tissues (Figure 6), and that mitogenic stimulation can induce *PIM1* mRNA to a greater degree in TTP-deficient cells (Figure 7). Together, these data indicate that concomitant induction of TTP likely contributes to limiting the amplitude and duration of *PIM1* mRNA accumulation following mitogenic stimulation.

Recent ribonome-wide surveys of TTP-regulated mRNAs by large-scale RNP-IP [31], or differential mRNA levels [32] or stability [33] in cells expressing or lacking functional TTP have identified several hundred transcripts that may bind and/or be regulated by this protein. This putative TTP substrate population includes many mRNAs that encode regulators of cell proliferation and survival including cyclin G2, interleukins -10 and -15, and the polo-like kinases Plk2 and Plk3. Other known mRNA substrates of TTP encode factors that promote angiogenesis and tumor metastasis like vascular endothelial growth factor [34], [54] and urokinase plasminogen activator [32], as well as a diverse collection of inflammatory mediators including TNFα and cyclooxygenase 2 [55], [56]. As such, the mRNA-destabilizing activity of TTP likely serves as a general mechanism to limit levels of many transcripts whose uncontrolled expression can elicit severe pathological consequences. However, the ordered activation of positive (transcriptional induction, mRNA stabilization) and negative (TTP expression) regulatory mechanisms influencing *PIM1* mRNA following mitogenic stimulation characterized in this work highlights an expanded role for TTP in controlling expression of its mRNA targets. By coordinating the induction of TTP along with TTP substrate mRNAs in response to specific stimuli, cells may buffer perturbations in gene regulatory networks by limiting the extent and duration of target mRNA accumulation. The utility of regulated mRNA decay in limiting acute mRNA induction following inflammatory stress was recently highlighted in a survey of transcript levels and stability in lipopolysaccharide-stimulated bone marrow-derived macrophages [57]. Here, brief (30 min) lipopolysaccharide exposure stabilized a diverse array of ARE-containing transcripts; however, a subset of these mRNAs including those encoding endothelin 1, TNFα, the chemokine CXCL1, and even TTP itself were again rapidly degraded 6 h post-stimulation. This study suggests that post-transcriptional mechanisms targeting AREs may exert a limiting influence on the expression of many genes.

Although TTP expression is induced by selected mitogenic and inflammatory stimuli concomitant with activated transcription of some TTP substrate mRNAs including *PIM1* (discussed above), few details are available regarding the mechanisms responsible for coordinated transcription from these genes. For example, increased TTP expression in lipopolysaccharide-stimulated cultured macrophages requires p38^MAPK^ [58], while serum induction of TTP in fibroblast models was strongly but not completely dependent on an intronic sequence element that bound the transcription factor Sp1 [59]. By contrast, neither of these mechanisms has yet been implicated in the regulation of the *PIM1* gene, although prolactin activates its transcription in a lymphoma model through several proximal upstream promoter elements [22], and also requires activation of the Akt kinase [60]. However, a recent ribonome-scale survey of epidermal growth factor-stimulated genes in HeLa cells showed that induction of TTP mRNA coincided with expression of several transcription factors including junB and ATF3, suggesting that an AP-1-based transcription circuit could be responsible for coordinating these events [61]. Elucidating the molecular mechanisms responsible for coordinating transcription of TTP and its target mRNAs thus remains an intriguing topic for future study.

Data presented in this work show that TTP destabilizes *PIM1* mRNA through interactions with an ARE sequence in the *PIM1* mRNA 3′UTR, and that this regulatory mechanism suppresses *PIM1* expression 4 hours following mitogenic stimulation (Figure 1 and Table 1). However, in quiescent cells *PIM1* mRNA also decayed rapidly but was dramatically stabilized shortly following exposure to serum+TPA, all in the absence of detectable TTP protein (Figure 3). These observations prompt another interesting question, in that the mechanism(s) responsible for initial stabilization of *PIM1* mRNA following mitogen exposure remain unknown. Some results from this study suggest that constitutive decay of *PIM1* mRNA may also be mediated by its ARE domain, since the βG reporter mRNA lacking the *PIM1* ARE (ΔARE) was stabilized >2-fold *versus* reporter transcripts containing the complete *PIM1* 3′UTR (*p* = 0.0056) or the ARE alone (*p* = 0.0039), even in the absence of TTP (Table 2). Accordingly, a likely model is that an alternative ARE-binding activity is responsible for the rapid decay of *PIM1* mRNA in unstimulated cells, which may be inactivated or displaced shortly following mitogenic simulation. Over 20 different factors have been shown to bind AREs, although the functional significance of these interactions has not been resolved in most cases [41], [42]. However, recent studies on the regulation of selected ARE-binding proteins suggest some potential candidates. AUF1 is a family of four related proteins generated by alternative splicing from a common pre-mRNA [62]. Each isoform is capable of binding ARE sequences with varying degrees of affinity [63], but the major cytoplasmic isoforms, termed p37^AUF1^ and p40^AUF1^, are most closely associated with destabilization of mRNA substrates [64], [65]. In unstimulated THP-1 monocytes, polysome-associated p40^AUF1^ is phosphorylated on Ser83 and Ser87. However, stimulation of THP-1 cells with TPA induces rapid dephosphorylation of p40^AUF1^ concomitant with stabilization of mRNA targets [66]. A second potential *trans*-regulator of *PIM1* mRNA decay is HuR. This ubiquitously expressed protein stabilizes a wide variety of ARE-containing mRNAs [67]–[69] by forming cooperative oligomeric complexes on RNA substrates [70], [71]. HuR is principally nuclear, but stabilizes mRNA targets when translocated to the cytoplasm [72]. The nuclear-cytoplasmic distribution of HuR is regulated by several intracellular signaling pathways [73]–[75], including some associated with mitogenic stimulation like selected isoforms of protein kinase C [76], [77] and the p38^MAPK^ pathway [78].

While our study demonstrates that post-induction destabilization of *PIM1* mRNA is associated with accumulation of TTP levels, additional mechanisms may “fine-tune” temporal control of gene activation through ARE-directed mRNA decay. First, TTP itself may be regulated by phosphorylation *via* the p38^MAPK^-activated kinase MK2 [79], which promotes association with cytoplasmic 14-3-3 proteins [80], [81]. Second, TTP expression is also temporally regulated, as indicated by decreases in TTP protein levels 12 hours after stimulation of HeLa cells with serum+TPA, or as early as 4 to 6 hours post-stimulation in MDA-MB-231 cells (Figure 3B). It is likely that several mechanisms contribute to post-induction suppression of TTP levels, including protein turnover through proteasome pathway [79], [82], and ARE-directed destabilization of TTP mRNA, which can be enhanced by TTP in a negative feedback loop [83]. Third, gene regulatory effects of transiently increasing TTP levels are unlikely to be limited solely to mRNA decay, since many mRNAs containing ARE motifs encode transcriptional regulators [84]. By destabilizing some of these transcripts, TTP can suppress levels of their encoded protein products, and hence the ability of these factors to regulate transcription of their target genes. Finally, TTP is one of a large population of cellular factors competing for many ARE-containing transcripts (described above). Gene-specific consequences of mitogenic or other stimuli on post-transcriptional control of gene expression will thus be influenced by competition or cooperation among diverse ARE-binding proteins, among which many may be subject to stimulus-dependent regulation of expression and/or activity.

## Supporting Information

Table S1 **qRT-PCR primer sets used in this study.** Forward and reverse amplification primers are listed for all mRNAs quantified by qRT-PCR. For mRNAs quantified using multiplex qRT-PCR reactions, TaqMan probe sequences and associated dye/quencher pairs are also included.
(DOC)
