# Supplementary material for: Coordinated Expression of Tristetraprolin Post-Transcriptionally Attenuates Mitogenic Induction of the Oncogenic Ser/Thr Kinase Pim-1
Source: PLoS One. 2012 Mar 8;7(3):e33194. doi: 10.1371/journal.pone.0033194 (PMC3297641; doi:10.1371/journal.pone.0033194)
Supplement: Table S1 — qRT-PCR primer sets used in this study. Forward and reverse amplification primers are listed for all mRNAs quantified by qRT-PCR. For mRNAs quantified using multiplex qRT-PCR reactions, TaqMan probe sequences and associated dye/quencher pairs are also included. (DOC) [file pone.0033194.s001.doc]

**Supplemental Table S1: qRT-PCR primer sets used in this study**

| DNA oligo | sequence (5’ to 3’) |
| --- | --- |
| *human PIM1 SYBR primer set* | |
| forward | GACCTGCACGCCACCAAG |
| reverse | CGGCAAGTTGTCGGAGACG |
|  |  |
| *human TTP SYBR primer set* | |
| forward | GGCCAACCGTTACACCATG |
| reverse | GAGTCGGAGGGGCTCAGG |
|  |  |
| *human GAPDH SYBR primer set* | |
| forward | TGCACCACCAACTGCTTAGC |
| reverse | GGCATGGACTGTGGTCATGAG |
|  |  |
| *murine PIM1 SYBR primer set* | |
| forward | GACCTGCACGCCACCAAG |
| reverse | CGACGCGGATGCCAGAG |
|  |  |
| *murine GAPDH SYBR primer set* | |
| forward | ATGGTGAAGGTCGGTGTGAACG |
| reverse | CGCTCCTGGAAGATGGTGATGG |
|  |  |
| *β-globin reporter Taqman primer set* | |
| forward | GTGAACTGCACTGTGACAAGC |
| reverse | ATGAGTAGACAGCACAATAACCAG |
| probe | Fl-CGTTGCCCAGGAGCCTGAAGTTCTCA-BHQ1*^a^* |
|  |  |
| *EGFP Taqman primer set* | |
| forward | GCGACACCCTGGTGAACC |
| reverse | GATGTTGTGGCGGATCTTGAAG |
| probe | TxR-CACCTTGATGCCGTTCTTCTGCTTGTCG-BHQ2*^a^* |

*^a^*Abbreviations are: BHQ1, Black Hole Quencher 1; BHQ2, Black Hole Quencher 2; Fl, fluorescein; TxR, Texas Red.
